# Gummies: From Confectionery to Functional Hydrogel-Based Bioactive Delivery Systems

**DOI:** 10.3390/gels12090841

**Published:** 2026-09-15

**Authors:** Max Vásquez-Senador, Indira Pérez-Bermúdez, Patricio Orellana-Palma, Guillermo Petzold

**Affiliations:** 1 Departamento de Ingeniería en Alimentos, Facultad de Ciencias de la Salud y de los Alimentos, Universidad del Bío-Bío, Campus Fernando May, Av. Andrés Bello 720, Chillán 3780000, Chile; max.vasquez2501@alumnos.ubiobio.cl; 2 Centro de Estudios en Alimentos Procesados (CEAP), Campus Lircay, Talca 3480094, Chile; iperez@ceap.cl; 3 Escuela de Nutrición y Dietética, Centro Integrativo de Biología y Química Aplicada (CIBQA), Facultad de Ciencias de la Salud, Universidad Bernardo O’Higgins, Calle General Gana 1702, Santiago 8370854, Chile

**Keywords:** functional gummies, hydrogels, hydrocolloids, sweetening systems, bioactive compounds, emerging technologies

## Abstract

Gummies have evolved from traditional confectionery products into hydrogel-based systems capable of incorporating and releasing bioactive compounds. This review examines the main formulation and technological factors governing their structure and functionality, with an emphasis on the use of hydrocolloids, alternative sweetening systems, and molecular interactions, the incorporation of bioactives, and the recent use of emerging technologies in their production. The literature was retrieved from Scopus, Web of Science, ScienceDirect, and Google Scholar, encompassing studies published between 2005 and August 2026. The literature shows that the type of hydrocolloid and the sweetener composition strongly influence gel formation, water mobility, hydrogen bonding, crystallization, texture, and storage stability. Plant byproducts, extracts, and marine-derived ingredients provide additional sources of bioactive compounds, while microencapsulation, nanoencapsulation, high-pressure processing, cryoconcentration, ultrasound, and 3D printing offer opportunities to enhance their protection and release. However, interactions between bioactive compounds and the gel matrix can affect texture, sensory acceptance, stability, and bioaccessibility. Therefore, developing functional gummies requires a comprehensive understanding of formulation, hydrogel structure, processing conditions, and bioactive stability to obtain products with suitable technological, sensory, and functional properties.

## 1. Introduction

**Figure 1:**
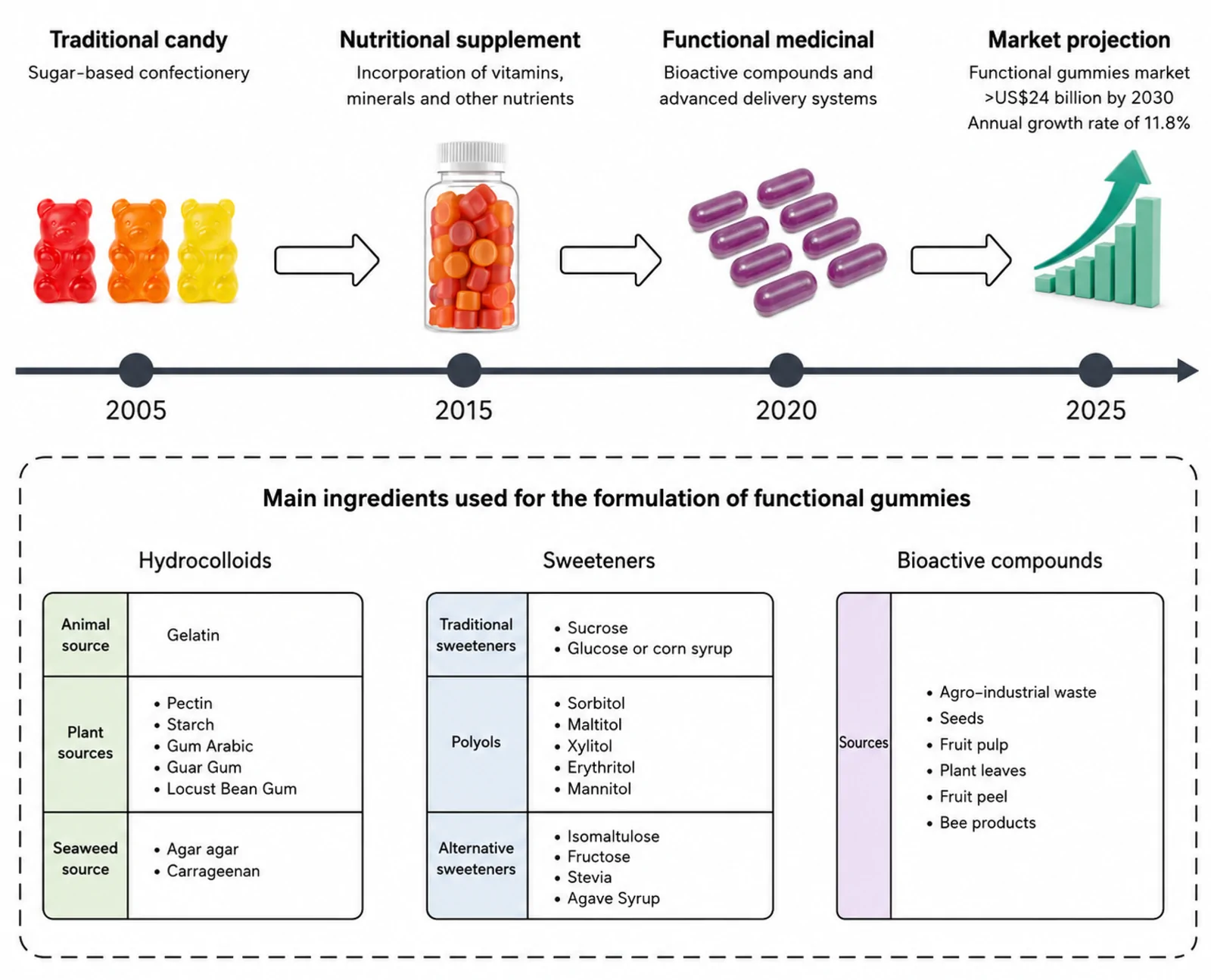
Evolution of gummy products from conventional sugar-based confectionery to functional, nutraceutical, and bioactive delivery systems (2005–2025), including the main ingredients used for the formulation of gummies on the timeline.

Gummies are among the most popular confectionery products across all age groups due to their pleasant sensory characteristics, soft texture, wide variety of flavors and colors, and different formulations [1]. Traditionally, gummies are produced using hydrocolloids as gelling agents combined with sweeteners (sucrose and glucose syrup), food acids, flavoring agents and colorants [2,3]. However, these products have evolved considerably in response to the growing demand for novel sensory experiences, as well as health-oriented foods [4]. Accordingly, their soft texture, high consumer acceptance, convenient dosage format, and formulation versatility make gummies attractive matrices for the incorporation of health-promoting compounds. In this context, gummies have attracted considerable scientific interest due to their potential application as hydrogel-based delivery systems for various bioactive compounds, including vitamins, polyphenols, peptides, minerals, probiotics, and nutraceutical ingredients, enhancing their potential as functional products with health benefits for consumers [5,6]. Hence, gummy development has progressively shifted from conventional sugar-based confectionery toward functional, nutraceutical, and even medicinal delivery systems. Specifically, the 21st century has witnessed growing consumer awareness of the health benefits of foods, leading to increased interest in bioactive compounds (functional molecules naturally present in foods, plants, and agro-industrial byproducts that exert beneficial biological activities, including antioxidant, antimicrobial, and anti-inflammatory effects, among others, favoring the consumption of foods and food by-products [7,8,9,10]. This trend has promoted a remarkable re-engineering process in gummy formulations through the incorporation of novel ingredients and the application of both traditional and emerging technologies [11]. As a result, gummy formulations have diversified substantially in terms of ingredient selection, including alternative sweeteners, plant-based hydrocolloids, and natural bioactive compounds derived from agro-industrial byproducts [12,13,14]. These advances in the production of functional gummies have contributed to the rapid expansion of this sector, since the global market projected to exceed US$24 billion by 2030, reflecting the rapid expansion of this sector, with an annual growth rate of 11.8% (Figure 1) [15].

Despite these advances, the development of functional gummies still faces important challenges associated with physicochemical stability, texture properties, sensory acceptance, and shelf-life preservation. These limitations are strongly influenced by the ingredients (the origin and concentration of hydrocolloids, the use of sweeteners or sugar, the type of flavoring and coloring agents, and the incorporation of bioactive compounds, among others) [16]. Furthermore, the replacement of synthetic additives with natural ingredients from fruit and/or plant extracts introduces additional formulation complexities, particularly concerning stability during storage and the organoleptic qualities of the final product [17]. Therefore, the rapid growth of the scientific literature and the increasing complexity of gummy formulations and applications suggest that considerable research will be carried out in the coming years. In this context, the present review focuses on functional gummies as hydrogel-based delivery systems, emphasizing the relationship between ingredient composition, processing conditions, and final product performance. Additionally, this review integrates hydrocolloid interactions, alternative sweetening systems, incorporation of bioactive compounds, encapsulation systems, sustainable raw materials, nutraceutical products, specialized delivery applications and emerging processing technologies into a structure–function framework, highlighting the impact of these factors on physicochemical stability, texture properties, sensory quality, and functionality. Moreover, particular attention is given to the current limitations, formulation trade-offs, and future challenges associated with the development of next-generation functional gummy products. Table 1 summarizes the main review articles published between 2015 and 2026 related to functional gummies and closely associated research areas.

As summarized in Table 1, current reviews reflect the increasing diversification of research on functional gummies, focusing on specific ingredients, delivery systems, health applications, or formulation strategies, including hydrocolloids of animal, plant, and seaweed origin; microencapsulation techniques for bioactives and prebiotics, the use of medicinal plant extracts, and nutraceutical formulations. While these contributions provide information on specific areas, an integrated analysis linking hydrocolloid selection, sweetening systems, bioactive incorporation, hydrogel structure–function relationships, physicochemical and sensory properties, and emerging processing technologies remains limited. Therefore, the novelty of this review lies in its analysis of the transition of gummies from conventional confectionery products to hydrogel-based bioactive delivery systems within a unified framework.

## 2. Gummy Matrix: The Role of Hydrocolloids

### 2.1. Animal-Based Proteins

Traditional gummy candies are typically formulated with high amounts of sucrose and glucose syrup combined with a gelling agent, most commonly gelatin [25], which is generally obtained through the partial hydrolysis of collagen, the most abundant structural protein present in animal connective tissues such as skin, bones, tendons, and cartilage [26,27,28,29]. Specifically, gelatin is widely used in the confectionery industry due to its excellent gelling, stabilizing, foaming, and texturizing properties [30]. These characteristics are strongly influenced by the high-molecular-weight distribution and length of the polypeptide chains, which depend on processing conditions such as extraction time, temperature, and interactions with other ingredients, including sugars and polyphenols [31]. However, despite its technological advantages, the use of gelatin in the development of healthier gummy products presents two main challenges: (i) as an animal-derived ingredient obtained mainly from porcine, bovine, fish, and poultry, among others, it excludes vegan and vegetarian consumers, and similarly, gelatin-based gummies pose limitations in markets governed by religious dietary requirements, such as Halal and Kosher certifications [32], and (ii) gelatin exhibits thermoreversible behavior, whereby if the final product is exposed to elevated temperatures, the matrix undergoes considerable melting, and even, it can return to a liquid phase. Hence, storage conditions must be carefully controlled to preserve the product integrity, particularly in warm climates or during summer season [33]. Hence, current research efforts are focused on improving the functionality of animal-derived gelatin at the molecular level, optimizing the structural organization of the polymeric network and its rheological properties to optimize the gel strength and thermal stability. These approaches seek to overcome the inherent limitations of gelatin, maintaining its desirable sensory and technological attributes [34,35,36,37].

### 2.2. Plant-Based Polysaccharides

Growing consumer demand for vegan, vegetarian, and clean-label products has stimulated increasing interest in plant-based polysaccharides as alternatives to animal-derived gelatin. These hydrocolloids provide diverse gelling mechanisms and textural properties, making them suitable for the development of gummies. Among the most commonly used plant-based polysaccharides are pectin, starches, and seaweed-derived hydrocolloids, each of which offers distinct technological advantages and limitations.

#### 2.2.1. Pectin

As a vegan ingredient, pectin is a non-toxic complex polysaccharide widely used in the food and pharmaceutical industries [38,39]. Its chemical structure consists primarily of galacturonic acid units linked by α-(1→4) glycosidic bonds [40]. Based on its degree of esterification, pectin is classified into high-methoxyl pectin (HMP) and low-methoxyl pectin (LMP) [41], with HMP forming gels under acidic conditions and in the presence of relatively high sugar concentrations, whereas LMP forms gels through ionic cross-linking with divalent cations, particularly calcium ions [42]. Compared with gelatin, pectin exhibits a different gelling profile, since it produces gummies with lower elasticity and a shorter bite, resulting in a distinct sensory experience. However, pectin may require higher sugar concentrations to achieve the desired sweetness and flavor [43]. Several studies have reported that a high concentration of pectin in the formulation of gummies can reduce chewiness, and consequently, decrease consumer acceptability [44]. In addition, LMP presents a network structure with relatively large pore sizes, and it contributes to lower elasticity, but an increase in the concentration of LMP generally results in a firmer texture [45]. Therefore, due to its plant origin, versatility, and compatibility with vegan formulations, pectin has become one of the most widely used alternatives to gelatin in the development of gummies and other confectionery products.

Similarly, soluble soybean polysaccharides (SSPS) are pectin-like polysaccharides obtained from soybean, particularly from the cell wall fraction of soybean-derived materials. Due to their structural characteristics and high content of soluble dietary fiber, SSPS have attracted increasing interest as functional ingredients in food formulations [46]. Their amphiphilic properties and ability to interact with proteins and other food components provide useful functional properties, including emulsification, stabilization, and water retention [47]. In addition, SSPS have been incorporated into confectionery and gummy formulations as plant-based ingredients, contributing to dietary fiber enrichment and potentially enhancing the functional and nutritional value of these products [48,49,50]. Therefore, SSPS represent a promising alternative ingredient for the development of vegan gummies and other functional confectionery products, particularly when the objective is to combine textural functionality with dietary fiber enrichment.

#### 2.2.2. Starch

Starch is recognized as one of the most abundant plant-derived polysaccharides and it is widely used in the food industry as a thickening, stabilizing, and structuring agent [51,52]. It is mainly composed of two glucose polymers, amylose and amylopectin, and their relative proportions influence the gelatinization behavior, gel formation, and the final textural properties of food products [53]. Specifically, the gelling properties are associated with the gelatinization process, in which starch granules absorb water, swell, and partially lose their crystalline structure upon heating, and later, as the system cools, the leached polymers reorganize and form a three-dimensional network capable of retaining water, providing structural integrity to the hydrogel matrix [54]. Commercial starch used in gummy formulations is commonly obtained from different food sources such as corn, cassava, potato, and rice, among others, providing a plant-based alternative to gelatin, and in turn, contributing to the development of gummy products with desirable texture, stability, and mouthfeel [13]. In gummy formulations, starch acts as an important structuring agent, optimizing water retention and modifying textural characteristics. Partial replacement of gelatin with cornstarch has been reported to increase adhesiveness and to enhance mechanical properties of the matrix [55]. Similarly, acid-modified cassava starch provides hardness and chewiness values comparable to those obtained with conventional corn starch, and in turn, it maintains high sensory acceptability [56]. Despite their advantages, starch-based gels generally exhibit lower elasticity than gelatin-based products and it may be susceptible to retrogradation during storage, altering their texture over time [57]. Moreover, excessive starch concentrations may significantly increase hardness and chewiness, resulting in overly firm products and reduced consumer acceptance [43]. Hence, their low cost, wide availability, plant origin, and compatibility with vegan formulations make starches attractive ingredients for the development of functional gummy products [58].

#### 2.2.3. Seaweed

Seaweeds constitute an abundant and renewable source of polysaccharides, with the most important seaweed-derived hydrocolloids being agar, alginate, and carrageenan, which are extracted from red and brown algae. In addition, these provide essential vitamins, proteins, lipids, and minerals [59]. Specifically, each polysaccharide exhibits distinct chemical structures, determining its functional properties. Agar is primarily composed of agarose and agaropectin, alginate consists of β-D-mannuronic acid and α-L-guluronic acid, while carrageenan is formed by sulfated galactan chains with varying degrees of sulfation [18]. Accordingly, based on their chemical composition and molecular arrangement, these hydrocolloids present different gelation mechanisms, with agar forms thermoreversible gels upon cooling [60], alginate produces gels through ionic cross-linking with divalent cations (calcium) [61], and carrageenan forms gels through interactions with specific ions and polysaccharide chains [62]. These mechanisms enable the formation of three-dimensional networks capable of retaining water and providing structural stability to hydrogel-based systems.

From technological and functional perspectives, seaweed hydrocolloids offer excellent water-holding capacity, thermal properties, viscosity control, and textural modification [63]. Consequently, these hydrocolloids can serve as alternatives to animal-derived gelatin in gummy formulations [64], contributing to products with desirable texture, firmness, elasticity, and shelf-life stability, supporting vegan and clean-label claims [65]. Despite these advantages, the development of seaweed-derived gummies may present several challenges such as sensory limitations due to the potential presence of marine off-flavors, undesirable colors and changes in texture, affecting the consumer acceptance [59]. In addition, the formulation complexity arises from the need to optimize hydrocolloid concentrations and their interactions with other ingredients to achieve desirable gel characteristics [66]. Regulatory challenges are also important, since the final approval, purity specifications, labeling requirements, and maximum permitted use levels of seaweed-derived ingredients may vary among countries and regions. Furthermore, the concentration of contaminants such as heavy metals, iodine, and arsenic requires strict quality control and compliance with food safety regulations. These factors may limit the commercialization of seaweed-based products, including gummies, and thus, the complexity of their industrial implementation could increase significantly [67]. However, the origin, sustainability, and versatile technological and functional properties of these hydrocolloids have promoted multiple applications of seaweed-derived ingredients in different areas such as confectionery, nutraceutical, pharmaceutical, and biomaterials industries [68,69].

Furthermore, natural and emerging seaweed polysaccharides such as fucoidan and ulvan have attracted growing attention due to their bioactive properties and versatile chemical functionalities [70]. Thus, these polysaccharides exhibit limited gel-forming ability on their own, but they can be mixed with other hydrocolloids or suitable cross-linking agents to form multifaceted biomaterials, improving the control over gelation behavior and the mechanical properties of the resulting matrices, making them promising materials for advanced food and non-food applications [71,72]. Specifically, fucoidan, a natural sulfated polysaccharide, can be used for the development of hydrogels under certain conditions. For example, Afrin et al. [73] used a hydrazone crosslinked fucoidan (hcFu) hydrogel for tissue engineering applications, in which fucoidan was chemically modified, resulting in a hydrogel with interesting viscoelasticity, self-healing, extrudability, injectability, and excellent capacity to support cell growth. Moreover, Wang et al. [74] mixed κ-carrageenan with fucoidan at different concentrations to form a stable gel blend, highlighting the formation of hydrogen bonds between κ-carrageenan and fucoidan chains, and thus, the final biomaterial showed high water retention, resistance, gelling capacity and thermal stability. In the same way, ulvan (a water-soluble anionic polysaccharide) has been used to develop biomaterials with other polysaccharides due to its ionic characteristics [75]. Mariia et al. [76] used chitosan to reinforce the properties of an ulvan hydrogel, and thus, it allowed the development of a novel chitosan-ulvan hydrogel as a wound dressing material, with a microstructure suitable for the transport of compounds, high mechanical resistance, and thermal and swelling stability. Furthermore, Hwang et al. [77] added alginate to an ulvan solution to develop an alginate–ulvan hydrogel using ionic crosslinking for medical applications, revealing interconnected porous structures, which facilitated the transport of essential nutrients, with high Young’s modulus, hardness, and structural stability. Hence, these natural biopolymers represent promising candidates for the development of controlled-release systems and next-generation functional gummy formulations enriched with bioactive compounds.

Overall, the abundance, sustainability, cost-effectiveness, and versatile functional properties of seaweed-derived polysaccharides make them promising alternatives to conventional animal-based gelling agents, highlighting their potential to support the development of innovative, clean-label, and value-added functional gummy products.

#### 2.2.4. Other Hydrocolloids

In addition to the conventional gelling agents previously described, other hydrocolloids have emerged as promising edible polymers for the development of gummy formulations. These materials provide distinct gelation mechanisms, offering opportunities to tailor texture, stability, and bioactive compound delivery according to specific formulations. This reflects the continuous search for innovative, sustainable, and clean-label alternatives for the development of next-generation functional gummies.

Gum arabic is used in the confectionery industry as a stabilizer and thickener, since it prevents sugar crystallization and improves the texture properties, minimizing tooth adhesion [78]. Additionally, it acts as an excellent microencapsulating agent, protecting volatile and bioactive compounds due to its high solubility and low viscosity [79]. Furthermore, this hydrocolloid is considered a prebiotic dietary fiber and its low calorie content makes it a key ingredient for the development of healthy and dietetic gummies [80]. Meanwhile, guar gum acts as an effective thickening agent and it contributes to the modification of textural properties in biomaterial matrices [81]. In combination with agar-agar, the resulting matrix exhibits enhanced elasticity and chewiness, which can be attributed to intermolecular interactions involving the galactose side chains of guar gum, resulting in a cohesive and resilient network comparable to the sample obtained with conventional gelatin [82]. Similarly, locust bean gum (a galactomannan extracted from the endosperm of locust bean seeds) possesses thickening, stabilizing and emulsifying characteristics. It can form a highly viscous solution at low temperatures and concentrations [83]. In conjunction with other polysaccharides, the matrix presents improved viscosity and gel strength. Recently, locust bean gum was combined with tilapia protein gelatin, resulting in acid gel gummies with high gel strength and melting temperature, and better structural properties compared with locust bean gum-based hydrogel, providing a theoretical basis for the development of innovative gummy products and broader applications in the food industry [84]. Table 2 summarizes recent studies on gummy formulations, highlighting the use of binary hydrocolloid systems, the incorporation of food by-products, and the effects of processing conditions on the microstructure and mechanical properties of the resulting matrices.

## 3. Sweetening Systems and Their Influence on Structural Stability

### 3.1. Conventional Sugars vs. Alternative Sweetening Systems

As was mentioned, gummies are typically formulated with hydrocolloids, sweeteners (sucrose and glucose syrup), and other ingredients (food acids, flavoring agents, and colorants) [2,3]. Specifically, the sweeteners are among the most important factors influencing consumer acceptance of gummies, since sweeteners play a dual technological role on the texture and flavor [88]. Hence, sugar influences the structural formation and stabilization of the hydrogel network, since it acts as a water-binding agent, reducing water activity by immobilizing free water molecules within the hydrogel network [89], lowering syneresis during storage at room temperature, and influencing the glass transition temperature [90]. Consequently, sweeteners directly affect firmness, elasticity, chewiness, stickiness, and shelf-life stability [91]. In this sense, an important property in gummies is the syneresis, a physical effect that refers to the loss of water from the gel matrix; sugar, in this aspect (and this is one of the characteristics that differentiates it from sucrose substitutes in gummies) binds the water in the gelatin matrix, reducing the unbound water and thus the syneresis [92].

However, excessive sugar consumption is associated with an increased risk of reactive oxygen species (ROS) production, leading to non-communicable diseases such as metabolic syndrome, diabetes, obesity, cancer, hypertension, dental caries, and cardiovascular disease [93,94,95]. In this context, the food industry is actively seeking alternative sweetening systems to develop healthier products, without affecting the molecular weight, hygroscopicity, water-binding capacity, crystallization behavior, and interactions with hydrocolloids, as well as sweetness release, moisture, and the sensory, texture, structural and stability properties of gummy candies [96].

In particular, polyols have attracted considerable attention as sucrose substitutes, since they provide sweetness and bulking capacity, maintaining the physicochemical and sensory properties of food products. In addition, these sweeteners may exhibit slower glycemic responses than sucrose, providing healthier options for people managing glucose homeostasis [97]. Studies have shown that polyols can modify the colloidal properties of gels, resulting in a strengthened gel network with increased yield stress. This effect has been attributed to the hydrophilicity of the polyols, which reduces the amount of water available for interaction with the colloid system, increasing the effective gelatin concentration in the low-acyl gellan gum gels [98].

Among sugar alcohols, xylitol is widely used in food formulations, since it exhibits high sweetening capacity, thermal stability, low caloric value (2.4 kcal/g) compared with normal carbohydrates (4.0 kcal/g), and cost-effectiveness [99]. These properties make it a suitable alternative to sucrose, particularly in the development of low-calorie formulations. Sasiluksananukul et al. [100] indicated that xylitol-based gummies showed comparable physicochemical properties and overall consumer acceptance to conventional gummies (with sucrose). In terms of the mechanical stability of the xylitol-based gummy matrix, it showed acceptable firmness during the first bite and adequate chewiness, highlighting that textural properties (hardness, gumminess, and chewiness) were closely associated with changes in moisture content and water-binding interactions within the gelatin network. These findings demonstrate that xylitol can partially or completely replace sucrose and preserve the structural and mechanical characteristics of gummies without markedly compromising the structural integrity of the hydrogel network.

### 3.2. Molecular Interactions Governing the Stability of Gummy Hydrogel Systems

The physicochemical properties of gummy hydrogels are governed by the selection of hydrocolloids and sweeteners but also by the molecular interactions established among polymers, sugars, polyols, and water molecules. These interactions determine the formation and stability of the three-dimensional network, directly influencing water retention, texture, mechanical strength, crystallization behavior, and shelf-life [101]. Consequently, understanding these molecular mechanisms is essential for designing functional gummy systems with improved structural stability and enhanced bioactive compound delivery.

#### 3.2.1. Molecular Interactions and Hydrogen Bonding

Hydrogel formation in gummy systems is primarily governed by non-covalent molecular interactions, particularly hydrogen bonding, together with polymer–polymer and polymer–water interactions. These interactions drive the association of biopolymer chains into a three-dimensional network that provides structural integrity and mechanical stability to gummy matrices [102,103]. Hydrogen bonds formed between hydroxyl, carboxyl, and amino groups stabilize the hydrogel network by promoting chain association and reducing molecular mobility, whereas polymer–water interactions regulate water immobilization, swelling behavior, and gel elasticity. Polymer–polymer interactions determine the density and connectivity of the hydrogel network, directly influencing hardness, cohesiveness, chewiness, and structural resistance. The incorporation of additional hydrocolloids may reinforce or weaken these intermolecular interactions depending on their chemical compatibility and concentration [104]. Consequently, the balance among hydrogen bonding, polymer–polymer interactions, and polymer–water interactions governs the physicochemical stability of gummy hydrogels and largely determines their suitability as matrices for the protection and controlled delivery of bioactive compounds [105].

#### 3.2.2. Role of Glucose Syrup and Dextrose Equivalent (DE)

Glucose syrup is one of the principal carbohydrate components in gummy formulations, serving as a sweetener, as well as a key structural ingredient that regulates gel formation, texture, and storage stability. Its functionality is largely determined by the DE, which reflects the degree of starch hydrolysis and consequently, the molecular weight distribution of glucose, maltose, oligosaccharides, and residual polysaccharides. Syrups with lower DE contain a greater proportion of high-molecular-weight polysaccharides, whereas higher-DE syrups are enriched in low-molecular-weight sugars, resulting in distinct physicochemical behaviors [106]. Variations in DE directly influence viscosity, water mobility, and polymer miscibility during gummy processing. Lower-DE glucose syrups generally increase viscosity due to their higher content of long-chain polysaccharides, which may affect phase stability, whereas higher-DE syrups improve polymer compatibility but may modify gelation kinetics and the final gel structure [107]. Furthermore, glucose syrup plays an essential role in controlling sucrose crystallization and reducing water activity, thereby contributing to the development of a homogeneous hydrogel network and extending product shelf life. However, excessive amounts of low-DE syrup may impair gel formation, leading to weaker gels, increased syneresis, and heterogeneous microstructures, highlighting the importance of selecting an appropriate DE according to the desired physicochemical and textural properties of gummy hydrogels [108].

#### 3.2.3. Polyols and Water Binding

Polyols, including sorbitol, maltitol, erythritol, isomalt, and xylitol, are widely incorporated into functional gummy formulations as partial or complete sucrose replacers, since they contribute to sweetness reduction, influencing the physicochemical stability of hydrogel systems. Their multiple hydroxyl groups enable extensive hydrogen-bond formation with hydrocolloids and water molecules, modifying the organization of the polymer network and improving water retention within the gel matrix [109]. Through their strong affinity for water, polyols may decrease water activity (a_w_) and reduce the mobility of free water, limiting moisture migration and potentially improving the microbial and physical stability of gummy products during storage. These interactions also influence water binding capacity and contribute to maintain a more homogeneous hydrogel network with reduced dehydration and syneresis [110]. In addition, polyols modify the thermal behavior of hydrogel systems by affecting the glass transition temperature (T_g_), molecular mobility, and polymer plasticization. Depending on their molecular size and chemical structure, polyols such as erythritol, xylitol, sorbitol, maltitol, and isomalt produce distinct effects on gel rigidity, elasticity, and textural stability, allowing the development of gummy matrices with improved mechanical properties and extended shelf life [82,110].

#### 3.2.4. Molecular Instability During Storage: Phase Separation, Syneresis and Crystallization

The structural stability of gummy hydrogels is highly dependent on the molecular compatibility among hydrocolloids, carbohydrates, and water. Thermodynamic incompatibility between polymeric components may induce phase separation, producing polymer-rich and sugar-rich domains that disrupt the continuity of the three-dimensional gel network and generate heterogeneous microstructures with reduced mechanical stability [106,108]. As phase separation progresses, the hydrogel may become more susceptible to syneresis, whereby polymer chain rearrangement promotes the gradual release of immobilized water during storage. The consequent reduction in water-holding capacity accelerates gel shrinkage, increases hardness, decreases elasticity, and compromises both the structural integrity and sensory quality of gummy products [106,110]. Another major deterioration mechanism is sucrose crystallization, which occurs when supersaturated sugar systems exhibit sufficient molecular mobility to initiate nucleation and crystal growth. Crystal formation disrupts the homogeneous hydrogel network, resulting in graining, opacity, undesirable textural changes, and ultimately a loss of product quality and shelf life. Recent studies have demonstrated that sucrose crystallization is governed by the combined effects of supersaturation, molecular mobility, glass transition temperature (T_g_), moisture content, and the sucrose-to-corn syrup ratio. Increasing corn syrup content and reducing molecular mobility can effectively delay nucleation and crystal growth, whereas appropriate interactions between gelatin and sucrose contribute to maintaining network integrity and storage stability [107,108].

#### 3.2.5. Implications for Texture and Shelf Life

**Figure 2:**
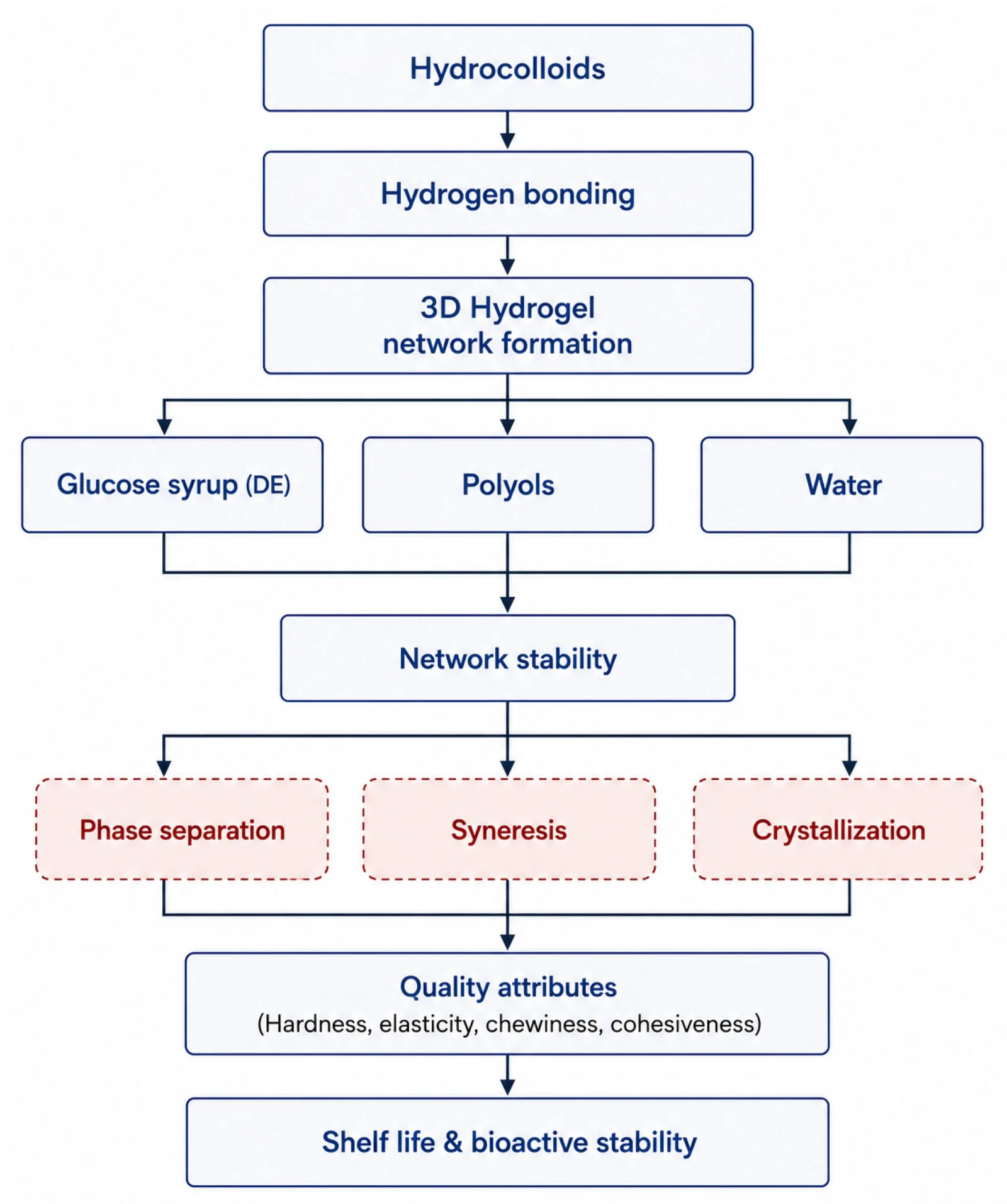
Proposed molecular interactions between hydrocolloids and sweetening systems governing the structural stability of gummy hydrogels. These interactions regulate hydrogel network formation, phase separation, syneresis, crystallization, texture, shelf life, and bioactive stability.

Collectively, the molecular interactions established among hydrocolloids, carbohydrates, polyols, and water determine the structural organization and long-term stability of gummy hydrogel systems. The balance between hydrogen bonding, polymer compatibility, and water immobilization governs the formation of a continuous three-dimensional network that directly influences hardness, elasticity, cohesiveness, springiness, and chewiness, while simultaneously controlling moisture retention and resistance to structural deformation [103,108]. During storage, changes in molecular mobility, water activity, and sugar composition may alter network organization, promoting phase separation, syneresis, and sucrose crystallization, which progressively compromise texture, appearance, and consumer acceptability. Consequently, the appropriate selection of hydrocolloids, glucose syrup characteristics (particularly dextrose equivalent), polyol type, and processing conditions is essential to preserve the amorphous gel structure and extend product shelf life [106,107]. From a formulation perspective, understanding these molecular mechanisms provides a rational basis for designing gummy hydrogels with tailored mechanical properties, improved storage stability, and enhanced protection of encapsulated bioactive compounds. This molecular approach is particularly relevant for the development of next-generation functional gummies, where hydrogel architecture must simultaneously ensure desirable sensory attributes, physicochemical stability, and efficient delivery of health-promoting ingredients [108,110]. Figure 2 summarizes the molecular mechanisms governing the formation, stability, and deterioration of gummy hydrogel systems. Hydrogen bonding promotes the formation of a stable three-dimensional network, whereas glucose syrup characteristics, polyols, and water collectively regulate network stability. During storage, molecular instability may trigger phase separation, syneresis, and crystallization, ultimately affecting texture and shelf life.

The molecular mechanisms summarized in Figure 2 are consistently reflected in experimental studies evaluating the effects of different sweetening systems on gummy hydrogel structure and mechanical performance. Recent investigations have demonstrated that conventional sugars and alternative sweeteners influence hydrogen bonding, water mobility, gel microstructure, and texture, ultimately determining the physicochemical stability of gummy matrices. In particular, plant-derived syrups have attracted attention for their functional properties, including their content of reducing sugars, which may enhance Maillard reaction potential and contribute to antioxidant activity [111]. Table 3 summarizes representative studies describing these relationships.

## 4. Sustainability and Incorporation of Bioactives into Gummies

The confectionery industry is increasingly embracing circular economy principles, since its production processes allow for the integration of fruit byproducts, which represent up to 35% of discarded raw materials. These byproducts can provide bioactive compounds and fiber, resulting in products with high nutritional value and potentially lower environmental impact. On the other hand, the incorporation of bioactives presents several challenges, including standardizing processing, ensuring the stability of compounds, and achieving consumer sensory acceptance [21]. This trend requires a comprehensive evaluation of functional confectionery, particularly studies that examine consumer perceptions, long-term health benefits, and regulatory aspects [123]. The use of natural preservatives and antioxidants, such as plant extracts (rosemary, green tea), vitamin C, and tocopherols, may extend the shelf life of foods without artificial additives. The incorporation of herbal extracts into confectionery products may provide bioactive effects, including anti-inflammatory activity, thus meeting consumer demand for natural formulations [124]. However, since functional foods contain bioactive compounds at specific concentrations or doses, they require appropriate safety and efficacy evaluation, including clinical studies when health claims are intended [125]. Some authors indicate that developing gummies with leaf extracts rich in phenolic compounds, flavonoids, and antioxidant activity may result in a bitter and astringent taste, reducing sensory acceptance; in response, the application of techniques such as spray-drying microencapsulation not only masks the bitter and astringent taste of the extract, improving product acceptability, but also provides a physical barrier that may limit direct interactions between the bioactive compounds and the protein or polysaccharide gel network. This protective effect contributes to preserve the structural integrity of the gel, improving the stability and controlled release of encapsulated compounds. Similarly, although the color of the gummies becomes darker as the concentration of the encapsulated extract increases, panelists may still assign acceptable sensory scores [126]. A recent study examined the interaction of gelatin with oregano essential oil, which improved disintegration time and reduced the swelling index without compromising the hydrocolloid’s tensile strength [127]. The incorporation of bioactive compounds directly affects the product’s sensory properties depending on their origin; for example, red beetroot provides betalain pigments and natural sugars that can function as colorants and sweeteners [128]. Similarly, guava can enrich the vitamin profile and provide pectin that contributes to gelation, as well as the fruit’s characteristic aroma. In this way, the plant-based sources may allow the replacement of synthetic additives, contributing to the stability and sensory acceptability of gummy products [129]. To obtain bioactive compounds for incorporation into gummies, different sources and extraction technologies have been investigated to obtain compounds with functional properties; some are summarized in Table 4.

## 5. Other Functional Additives in Gummies

Flower pollen is a complete food with high nutritional and bioactive value, notable for its content of phenols and flavonoids, as well as its antioxidant and anti-inflammatory activity, which has led to its incorporation into the formulation of functional gummies [180]. Ethanolic propolis extract has been microencapsulated by complex coacervation with chia mucilage and gelatin, resulting in high phenolic retention (15.36 mg gallic acid equivalents/g) and a remarkable antioxidant capacity (60.10 µmol TE/g) [181]. Similarly, the direct addition of dry green propolis extract has been shown to increase the antioxidant activity of gummies by up to 465%. In contrast, the sugars and fructans commonly used in gummy formulations do not possess antioxidant activity on their own [182]. Therefore, the incorporation of antioxidant-rich bulking agents, such as honey, may help enhance the antioxidant capacity of these products and notable antibacterial activity against *Streptococcus mutans*, *Staphylococcus aureus*, and *Escherichia coli* [183]. Furthermore, gummies containing honey and propolis have been reported to have an antioxidant capacity up to ten times greater than that of commercial products, retaining approximately 40% of this activity after in vitro digestion [184]. On the other hand, the incorporation of free propolis extract can negatively affect the texture and sensory acceptability of the gummies, increasing their hardness and generating undesirable flavors. However, both microencapsulation [181] and nanoencapsulation [185] allow the gel structure to be preserved, masking the bitter taste and maintaining antioxidant functionality without compromising the sensory quality of the final product.

Another interesting example of functional additives added to gummies is the use of tilapia skin-based formulations developed in combination with rice flour to improve texture, nutritional quality, and acceptability [186]. Similarly, sole gelatin has been transformed into selenium-containing nanoparticles, resulting in gummies with 72% antioxidant activity and improved texture [187]. Likewise, mackerel gelatin has been combined with soy milk to produce gummies with an optimal nutritional profile (16.20% protein) and high sensory acceptability [188].

## 6. Future Challenges

### 6.1. Sources

The valorization of agro-industrial byproducts (peels/waste), underutilized marine resources such as seaweed and the production of gelatin from fish skin represent a growing trend for obtaining bioactive compounds and gelling agents with functional potential [18,117]. Future research should explore additional plant-based sources, particularly fruit and vegetable processing byproducts such as citrus peels, grape pomace, apple pomace, pomegranate peels, tomato skins, and vegetable leaves, as well as their stabilization using technologies such as ionotropic gelation techniques and fermentation. These sources could provide valuable phenolic compounds, dietary fiber, pigments, and other bioactive constituents for the development of functional gummy formulations. An example of this approach is the production of vegan gummies using natural fruit juice and pulp [188].

### 6.2. Sweetener Interchange

Replacing sucrose with polyols (xylitol, isomalt, erythritol) or natural sweeteners (stevia, honey) in combination with plant-based hydrocolloids may reduce glycemic responses and improves the nutritional profile, but may affect the texture and consumer acceptance of the gummies. Also, the use of agro-industrial byproducts such as beetroot and agave to obtain syrups as bulking agents in gummy formulations is important, especially due to their compositional and functional properties [17,100,189].

### 6.3. Emerging Technologies

Microencapsulation and nanoencapsulation (using starch or gelatin nanoparticles) have proven effective in protecting bioactive compounds, masking undesirable flavors, and achieving controlled release [181,185,187].

High pressure processing (HPP) is driving the study of the behavior and stability (rheology) of gels, which can be used for the development of functional gummies, such as the application of HPP to the glucomannan polysaccharide from Konjac for gel optimization and pigment retention [190].

3D printing technology is key to the development of functional gummies, as it enables the incorporation and controlled release of bioactive compounds and drugs, improves sensory acceptability, and allows for texture adjustment based on consumer needs; functionally, the addition of peach gum polysaccharide (PGP) to gelatin gels has been shown to improve apparent viscosity and thermal stability, with printing accuracy greater than 90% and curcumin retention of 80% after gastrointestinal digestion [191]. In the medical field, 3D-printed semi-solid extrusion (SSE) was used to manufacture dual-action gummies containing isoniazid and pyridoxine, achieving 80% release within 30 min and 30 days of refrigerated stability, representing an advantageous alternative for personalized tuberculosis treatments [192]. Regarding texture, the addition of fruit powders and the use of polyols have been reported to improve the hardness and chewiness of the gels, increasing the gelation temperature (up to 42.3 °C) and enhancing the gel network through hydrogen bonding [98,193]. Despite the advantages in customization and texture control, 3D printing of gummies presents significant economic limitations, such as the high cost of equipment, low productivity per unit of time, and the need for specialized formulations.

On the other hand, cryoconcentration provides extracts rich in heat-labile compounds. Table 4 shows that most of the extracts incorporated into gummies come from thermal extraction processes, which may affect the activity of the bioactive compounds. Cryoconcentration is an emerging and promising technology for obtaining extracts rich in heat-labile compounds for functional gummies. This technology operates at low temperatures, preserving anthocyanins and polyphenols, and can achieve concentrations up to five times higher than those found in fresh sample. Extracts incorporated into commercial hydrogels have been shown to provide stability for up to 21 days [194].

### 6.4. Stability

The main challenge for texture and stability in gummies lies in balancing functionality and sensory quality; the incorporation of bioactive compounds or the substitution of sugars with polyols can alter the polymer network [98,195]. Future development focuses on designing matrices that use dual hydrocolloid systems (e.g., gelatin with hybrid carrageenans or agar with locust bean gum) that allow for modulating stiffness and elasticity, as well as mitigating syneresis problems [115,196]. Furthermore, the microencapsulation of bioactives is a key strategy to avoid unwanted interactions with the matrix [177,181], while the implementation of advanced rheological characterization techniques (such as large-amplitude oscillatory shear (LAOS) testing) will allow a deeper understanding of mouthfeel, texture perception, and flavor release [196,197].

### 6.5. Bioaccessibility

**Figure 3:**
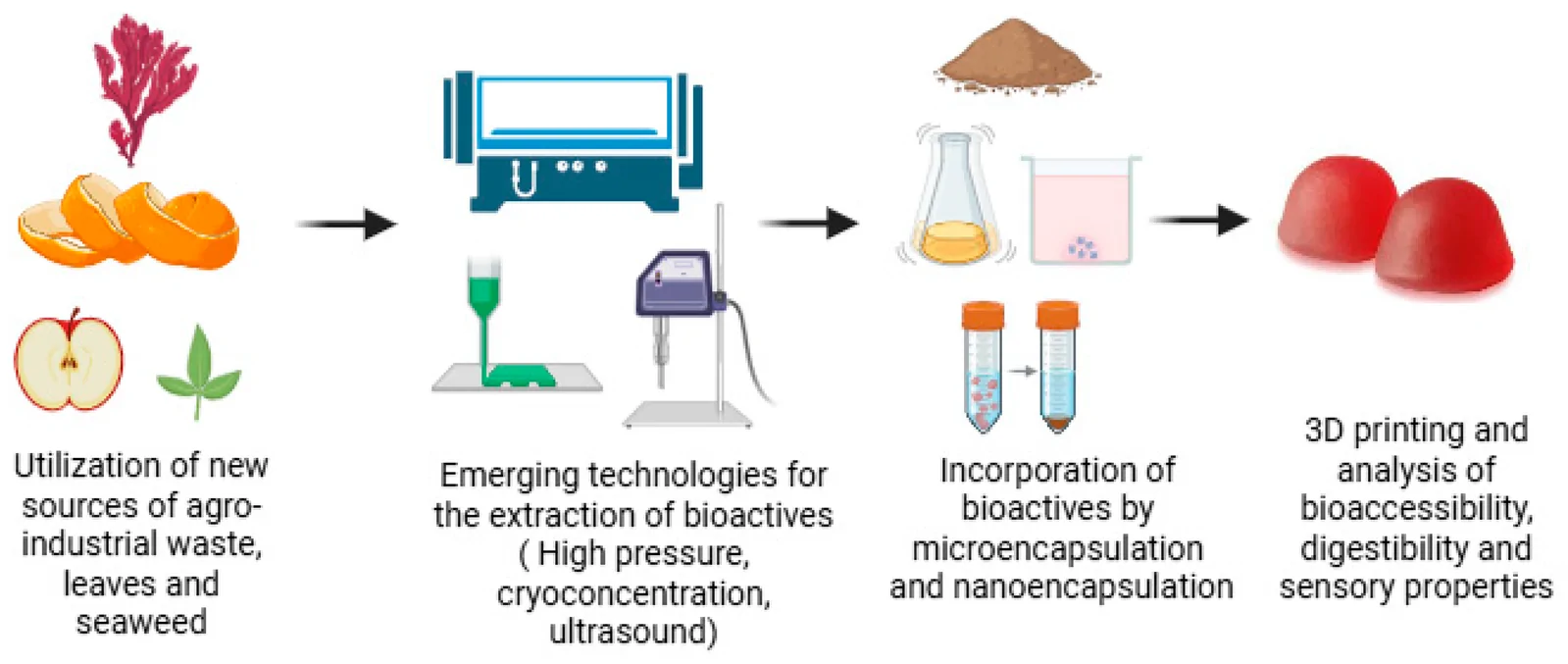
Summary of the main technological advances for the development of functional gummies, including the utilization of new sources (agro-industrial waste, leaves, seaweed), emerging technologies for bioactive extraction (high pressure, cryoconcentration, ultrasound), incorporation of bioactives via microencapsulation and nanoencapsulation, and 3D printing with analysis of bioaccessibility, digestibility, and sensory properties.

In the area of bioaccessibility, the main challenge lies in the low recovery of bioactive compounds after digestion and in unwanted interactions between the extracts and the gel matrix. Anthocyanin bioaccessibility percentages of less than 17% have been reported in gummies with blackcurrant pomace extract [132]. Other authors observed that the recovery of orange peel polyphenols in gummies was less than 45% of the added extract, which was attributed to possible thermal degradation or binding to gelatin proteins [138]. It has also been shown that the enzymatic extract of orange was not incorporated homogeneously into a pectin matrix, reducing the hardness of the gummies by up to 72% due to a suboptimal pH for gelation [137]. Furthermore, the viability of probiotics in gummies may be substantially reduced by 88% after intestinal digestion, with an estimated shelf life of 75 days at 4 °C [198]. Regarding the effects of functional gummies on gut microbiota, prolonged consumption of lutein-enriched gummies increased the Firmicutes/Bacteroidota ratio (a biomarker associated with obesity) [199]. Other authors confirmed in a clinical trial that pectin gummies were bioequivalent to capsules (with no significant improvement in bioavailability), unlike matrices with suspended lipids [200]. Future perspectives focus on optimizing the gel matrix to increase the release of hydrophobic compounds and protect probiotics, concentrating extracts through freeze-drying to increase the content of bioactives without compromising texture, and evaluating the long-term metabolic impact, especially in relation to the gut microbiota and metabolic health outcomes. Figure 3 summarizes the main technological advances for the production of functional gummies.

## 7. Conclusions

Gummies have evolved from conventional confectionery products into hydrogel-based systems for the incorporation and delivery of bioactive compounds. Their final properties depend on the interactions between hydrocolloids, sweeteners, water, and functional ingredients, which determine the structure, texture, stability, and retention of bioactive compounds. The use of alternative sweeteners, plant materials and agro-industrial byproducts, together with emerging processing technologies, offers opportunities but also presents challenges that need to be addressed to improve the nutritional and functional characteristics of gummies. Sensory acceptance, structural stability, and bioaccessibility remain key challenges in the development of functional gummies.

Current trends are primarily driven by consumer demand for low-sugar, natural, sustainable, and functional products, along with advancements in hydrogels and controlled-release technologies. Therefore, food engineering studies are needed to determine the stability, shelf life, and sensory acceptance of gummies, while interdisciplinary research with healthcare professionals is needed to better understand the interactions between the matrix and bioactive compounds and their effects on bioaccessibility and bioavailability. The proposed emerging technologies for gummy development and innovation should be further validated and scaled up, integrating technological, sensory, nutritional, and biological evidence to support the development of functional gummies with substantiated health benefits and appropriate regulatory support.

## 8. Materials and Methods

### 8.1. Review Design

This study was conducted as a structured narrative review. A narrative approach was selected because the review encompasses heterogeneous and interdisciplinary literature related to functional gummies, hydrogel-based food systems, natural polymers, sweeteners, bioactive compounds, delivery systems, food processing, and health-promoting properties. The purpose was therefore not to provide an exhaustive systematic inventory of the literature or to estimate pooled effects, but to critically integrate relevant technological, physicochemical, nutritional, and functional evidence on the development of functional gummy products.

The review was guided by four predefined questions concerning: (1) the main hydrogel-forming materials and natural polymers used in gummy and gummy-like food systems; (2) the role of sweeteners, formulation components, and processing conditions in determining the physicochemical and technological properties of functional gummies; (3) the incorporation, stabilization, and potential delivery of bioactive compounds in hydrogel-based gummy systems and their relationship to functional properties; and (4) the emerging technological approaches for the development of functional gummies, including advanced processing and 3D-printing technologies, as well as their potential for developing health-promoting food products.

### 8.2. Information Sources and Literature Search

The literature search was conducted using major scientific databases, including PubMed, Scopus, Web of Science Core Collection, SpringerLink, ScienceDirect, and Google Scholar. Searches were supplemented by backward citation searching of the reference lists of relevant publications and forward citation searching when necessary to identify subsequent studies addressing key concepts or technological approaches.

The search strategy combined terms related to several conceptual domains: (1) gummy and confectionery systems, including “gummies”, “gummy candies”, “functional gummies”, “functional confectionery”, and related terms; (2) hydrogels and food-grade polymers, including “hydrogel”, “food-grade hydrogels”, “hydrogel-based systems”, “natural polymer-based hydrogels”, “gels”, “hydrocolloids”, and related terms; (3) bioactive compounds and functional properties, including “bioactive compounds”, “antioxidants”, “functional foods”, “functional confectionery”, “health-promoting properties”, and related terms; and (4) delivery and emerging technologies, including “delivery systems”, “3D printing gummies”, “3D food printing”, and related terms. Search strings included combinations such as “gummies AND bioactive compounds”, “hydrogel-based systems AND functional confectionery”, “functional foods OR functional confectionery AND delivery systems”, and “3D printing gummies AND 3D food printing”, among others. Thus, search terms were combined using Boolean operators (AND/OR) and applied to refine and optimize the search results.

The search covered publications from January 2005 to August 2026. Publications available in English were prioritized. No geographical restriction was applied because the review considered international research on functional gummy and hydrogel-based food systems.

### 8.3. Eligibility and Literature Selection

Publications were considered eligible when they contributed directly to at least one of the predefined review domains and provided relevant technological, experimental, nutritional, physicochemical, or functional evidence. Eligible sources included original research articles with comprehensive experimental data, clear methodological descriptions, and scientifically robust findings, narrative and systematic reviews, and relevant conceptual or methodological studies addressing functional gummies, hydrogel-based food systems, natural polymers and hydrocolloids, sweeteners, bioactive compounds, delivery systems, processing technologies, or health-promoting properties. While, publications were excluded when they were not directly relevant to the development or characterization of gummy or hydrogel-based food systems, addressed the review topics only tangentially, or did not provide sufficient information, methodological transparency, and/or limited scientific evidence to contribute to the objectives of the review. Duplicate articles, non-scientific publications, conference abstracts, and conference proceedings without accessible full texts were excluded from the main literature synthesis.

The selection process involved an initial screening of titles and abstracts, followed by full-text assessment of potentially relevant publications. The selection was primarily based on thematic relevance and scientific contribution to the predefined review questions. Citation searching was used to identify additional relevant publications when necessary.

A total of 283 records were initially identified across the databases. The retrieved literature was subsequently screened for relevance to the predefined thematic areas of the review such as gummy formulations, the use of sweeteners, and the incorporation of different bioactive ingredients. Thus, after removal of 20 duplicates, 263 records were screened by title and abstract, and 40 publications were excluded at this stage. Subsequently, 223 full-text publications were assessed for eligibility, of which 16 were excluded, resulting in 207 publications included in the final narrative synthesis based on their relevance, scientific contribution, and availability of sufficient methodological information.

## Figures and Tables

**Table 1:** Recent review articles related to functional gummies and associated research topics published between 2015 and 2026.

Review Topic	Objective	Key Points/Sections	Reference
Healthy gummy candies	To review gummy candies as healthier food alternatives, emphasizing nutritional enhancement and their potential as functional foods	Functional ingredients; bioactive compounds; sugar substitutes; natural gelling agents; encapsulation; health benefits; consumer trends	[3]
Seaweed-based confectionery	To review the nutritional, technological, and sustainability potential of seaweed and seaweed-derived ingredients in confectionery products	Nutritional composition of seaweed; agar, alginate, and carrageenan; bioactive phytochemicals; applications in gummies, jelly candies, sensory, regulatory, and techno-logical challenges	[18]
CBD gummies and dietary supplements	To provide a comprehensive overview of cannabidiol (CBD) in dietary supplements, emphasizing formulation strategies, bioavailability, safety, and regulatory aspects	CBD chemistry; oral delivery systems; gummies; bioavailability; safety; regulations; future perspectives	[19]
Gum arabic–protein coacervation	Summarize advances in gum arabic–protein coacervation for food applications	Coacervation; encapsulation; bioactive delivery; protein modification; nonthermal technologies; food applications	[20]
Fruit pomace in confectionery	Summarize the use of fruit pomace in confectionery products	Pomace; bioactives; candies; gummies; preservation methods	[21]
Lactulose in functional foods	Review the history, mechanisms, technological properties, and food applications of lactulose	Lactulose; prebiotic effects; technological properties in confectionery and gummies	[22]
Vitamin microencapsulation	To systematically review the use of biopolymers and lipid-based coating materials for vitamin microencapsulation, emphasizing their effects on stability, controlled release, and food fortification	Microencapsulation techniques; coating materials (gums, alginate, gelatin, proteins, starches); controlled release; mathematical release models; fortified foods, including gummy candies	[23]
Medicinal gummies for gastrointestinal relief	To review medicinal gummies as a nutraceutical approach for gastrointestinal health	Herbal extracts; gastrointestinal health; gummy formulation; physicochemical properties; nutraceutical delivery	[24]

**Table 2:** Effect of hydrocolloid matrices and modifications on the structural and textural properties of functional gummies.

Study	Hydrocolloid Matrix	Focus/Modification	Key Structural and Textural Findings	Reference
Pectin/Gelatin gummy jelly	Gelatin + pectin	Hybrid network/Functional enrichment	The binary system (85:15 ratio) provides a balanced texture where gelatin ensures elasticity and pectin improves thermal stability. The combination results in a hardness of approximately 41 N and high consumer acceptance, effectively masking the functional extracts.	[4]
Sustainable Starch-based jellies	Pea starch vs. corn starch	Gelatin replacement/Amylose content impact	Pea starch (16–24%) creates a more rigid and less elastic network than gelatin. The amylose in pea starch pro-motes retrogradation, and results in greater hardness and gumminess, but lower resilience compared to corn starch and gelatin gummies.	[16]
Process-driven texture in model gels	Gelatin + sucrose/Glucose	Impact of mixing rate and addition temperature	High gelatin concentrations (10%) at a temperature of 55 °C improve hardness and chewiness. High mixing speed (1100 rpm) alters gumminess and elasticity	[25]
Emulsion-filled pectin gels	Pectin + gum arabic	Active fillers (oil droplets) and Vitamin delivery	The oil droplets increased the resistance of the pectinate network. The matrix protected the vitamins for 30 days.	[85]
Nondestructive TPA (Texture Profile Analysis) in gummy models	Gelatin + maltose	Small deformation TPA/Bloom value impact	High-bloom gelatin (240–260 g) creates a dense, interconnected porous network compared to low-bloom versions	[86]
Fucoxanthin-enriched seaweed gummy	κ-carrageenan + seaweed pulp	Marine-based functional matrix	Carrageenan and seaweed pulp (15%) improves hardness and chewiness of gummies	[87]

**Table 3:** Influence of sweetening systems on the structural and mechanical properties of gummy matrices.

Sweetening System	Hydrocolloid	Rheological and Mechanical/Textural Properties	Characterization Methods	Main Findings	Reference
Sucrose Glucose syrup *Garcinia atroviridis* puree	Salmon gelatin Pectin	Hardness, cohesiveness, adhesiveness, springiness, chewiness, gumminess	TPA	TPA demonstrated that increasing pectin and decreasing gelatin reduce hardness and chewiness of the gummies, while the opti-mized formulation achieved a textural bal-ance with adequate firmness and springiness, validated by high sensory acceptance.	[4]
Sucrose Glucose syrup Stevia Pistachio green shell extract	Gelatin Modified potato starch	Hardness, cohesiveness, springiness	TPA FTIR SEM	TPA showed that pistachio hull extract and the gelatin:starch ratio modify hardness, cohesiveness and springiness, allowing texture optimization. FTIR confirmed the physical entrapment of phenolic compounds in the matrix without new covalent interactions, and SEM revealed that the extract produces a denser and rougher structure, consistent with the higher hardness observed.	[12]
Maltol Erythritol Sorbitol Xylitol	Gelatin Low acyl gellan	Yield stress, viscosity, shear-thinning behavior, shear recovery, hardness, springiness, cohesiveness, gumminess, chewiness, and gel strength	FTIR SEM	Moderate polyol incorporation strengthened hydrogen bonding and excluded-volume effects. It increased gelation temperature, yield stress, viscosity and shear recovery performance, resulting in improved gel strength, hardness, gumminess, and chewiness. FTIR showed no new functional groups but stronger intermolecular hydrogen bonding. SEM revealed a denser and smoother gel network. Sorbitol and xylitol generated firmer gels than erythritol.	[98]
Eritritol	β-Glucan from Dictyophora rubrovalvata	Storage modulus (G’), viscosity, hardness, elasticity, cohesiveness, adhesiveness, chewiness, gumminess.	TPA FTIR SEM	Rheology confirmed that the microgel (β-glucan + erythritol) is a pseudoplastic fluid with higher viscosity and thermal stability; FTIR and SEM showed the formation of inter-helical hydrogen bonds that densify the gel network; and TPA demonstrated that the gummies present greater elasticity and chewability than commercial ones, with a softer and more resistant texture.	[110]
Maltitol Erythritol	Plant-based gummies	Hardness, springiness, cohesiveness, gumminess, chewiness, resilience	TPA	The replacement of sucrose with maltitol and erythritol in gummies increases the hardness, gumminess, and chewiness of the gel, which slows down glucose release during digestion, and combined with the low hydrolysis metabolism of polyols, significantly reduces the glycemic index compared to the control gummy, making them healthy options for glycemic control.	[112]
Mannitol Maltitol syrup	Gelatin	Hardness, springiness, cohesiveness, gumminess, chewiness, resilience	TPA	The substitution of sucrose with mannitol in gummy candies formulated with maltitol syrup increases hardness and reduces springiness due to mannitol’s low solubility and crystallization tendency, negatively affecting sensory quality, whereas soluble wheat fiber behaves similarly to sucrose, making it a better alternative for developing low-calorie gummies.	[113]
Sucrose Citric acid	Agar-agar Guar gum	Chewiness, gumminess, hardness, springiness, adhesiveness, firmness	TPA	The synergistic combination of agar-agar and guar gum as gelatin substitutes, together with sucrose and citric acid, enables the development of vegan gummies with texture similar to gelatin, which upon incorporation of turmeric and black pepper provide bioactive compounds and high antioxidant capacity, with excellent sensory acceptability.	[114]
Caster sugar High-fructose corn syrup	Kappa carrageenan Carboxymethylcellulose (CMC)	Hardness, adhesiveness, springiness, cohesiveness, gumminess, chewiness	FTIR SEM AFM	FTIR evidenced that carboxymethylcellulose at optimal concentration forms hydrogen bonds with carrageenan that strengthen the gel network; SEM revealed that this concentration produces a denser and more homogeneous network structure with similar-sized pores; and AFM confirmed that carboxymethylcellulose promotes side-by-side aggregation of carrageenan helices, forming a firmer and more stable structure.	[115]
Co-crystallized sucrose	Gelatin	Hardness, springiness, cohesiveness, gumminess, chewiness, adhesiveness, resilience	FTIR SEM DSC	FTIR confirmed the formation of intermolecular interactions between peppermint extract and sucrose during co-crystallization, SEM revealed a porous structure with cluster-like agglomerates that allow the entrapment of phenolic compounds, and DSC showed an increase in the melting point of sucrose indicating greater thermal stability of the co-crystallized powder.	[116]
Oligofructose Maltitol Stevia	Gelatin Microencapsulated pomegranate peel extract with maltodextrin	Firmness, hardness	SEM	SEM revealed that pomegranate peel extract microcapsules with maltodextrin are spherical particles with wrinkled surfaces that integrate into the gelatin matrix, and texture analysis demonstrated that their incorporation does not significantly affect firmness nor hardness of the gummies, although panelists perceived a slightly more acceptable texture in gummies with non-encapsulated extract.	[117]
Glucose syrup Sucrose	Gelatin Betaxanthin microcapsules	Hardness, adhesiveness, springiness, cohesiveness, gumminess, chewiness	FTIR SEM TPA	FTIR evidenced the formation of hydrogen bonds between betaxanthins and wall materials (mucilage or maltodextrin) without generating new functional groups; SEM revealed that microcapsules with mucilage have heterogeneous and rough particles that integrate into the gelatin matrix affecting texture; and TPA demonstrated that mucilage microcapsules reduce hardness and chewiness by acting as humectants, while increasing cohesiveness due to greater crosslinking with gelatin.	[118]
Jaggery apple juice	Agar-agar Kodo millet starch	Hardness, adhesiveness, cohesiveness, gumminess, chewiness	SEM TPA	SEM revealed that ultrasonication of Kodo millet starch produces granules with fissures and pores that improve their interaction with the agar-agar matrix, while TPA demonstrated that modified starch significantly increases hardness, gumminess and chewiness of the gummies, surpassing gelatin-based formulations.	[119]
Sodium saccharin Sodium cyclamate Mannitol	Gelatin Xanthan gum Trout collagen Hydroxyapatite Fish oil nanoemulsion	Hardness, adhesiveness, springiness, cohesiveness, gumminess, chewiness, resilience	TPA SEM FTIR	TPA analysis demonstrated that active ingredients reduce gummy hardness and gumminess while increasing springiness, facilitating their integration into the matrix due to the homogeneous, spherical, and porous morphology revealed by SEM inhydroxyapatite. Concurrently, FTIR assays confirmed the absence of unwanted chemical interactions with the gelatin, successfully preserving the structural integrity of the compounds.	[120]
Sorbitol Sugar Corn syrup	Pectin	Texture, shape, weight variation, dimension, swelling ratio, dispersion time, water activity	SEM FTIR	Sorbitol and pectin acted as sweetener and gelling agent, respectively, forming the base matrix of the gummies with elastic and non-sticky texture; SEM revealed that liposomal vesicles coated with the xyloglucan/trehalose/citric acid polymeric matrix were uniformly integrated into the pectin network, maintaining structural integrity; and FTIR confirmed that citric acid cross-linking reinforced the pectin matrix through ester bonds and hydrogen bonds, contributing to the firmness, springiness and textural stability of the gummies.	[121]
Xylitol Maltitol Inulin Fructooligosaccharides Xylooligosaccharides	Gelatin	Hardness, chewiness	TPA	Inulin as a sugar substitute alters the appearance and textural stability of gummies during storage.	[122]

**Table 4:** Bioactive sources, compounds, extraction technologies and functional effects in functional gummies.

Bioactive Source	Gummy Formulation	Experimental Model	Measured Outcomes	Main Findings	Reference
Jaban watermelon exocarp powder	Vegan gummies with 20–50% exocarp powder	Physicochemical, textural and sensory evaluation	Phenolics, flavonoids, antioxidant activity, texture, color, and sensory acceptance	35% exocarp with 0.75% citric acid and 0.5% agar showed the best sensory acceptance; increasing exocarp increased viscosity and hardness	[14]
Pomegranate peel extract	Jelly gummies with non-encapsulated or microencapsulated extract	Physicochemical, sensory and in vitro digestion	Polyphenols, punicalagin, ellagic acid, antioxidant activity and bioaccessibility	Microencapsulation markedly improved polyphenol bioaccessibility, particularly punicalagin	[117]
Red pitaya peel powde	Gummies with 0–3% citric acid	Physicochemical, antioxidant, and sensory evaluation	Phenolics, betacyanins, DPPH, pH, color, texture, and sensory acceptance	Citric acid increased antioxidant activity but reduced betacyanin content; 1% citric acid provided the best sensory acceptance	[130]
Grape pomace extracts	Alcohol-free wine-based jelly candies with Pinot noir or Chardonnay pomace extracts	Physicochemical, antioxidant, antidiabetic and sensory evaluation	Phenolics, antioxidant activity, α-glucosidase inhibition, sensory properties	Pinot noir gummies showed higher phenolics, antioxidant activity and α-glucosidase inhibition; Chardonnay gummies had better sensory acceptance due to lower astringency	[131]
Blackcurrant press cake extract	Functional gummies enriched with ultrasound-assisted extract	Chemical, cellular, in vitro digestion and sensory evaluation	Anthocyanins, antioxidant/anti-inflammatory activity, ROS, bioaccessibility and sensory acceptance	Gummies achieved 75% sensory acceptance; the extract reduced ROS in erythrocytes and THP-1 cells, but bioactive compound bioaccessibility decreased after digestion	[132]
Grape pomace extract	Color-rich gummies with anthocyanin-rich extracts obtained using natural eutectic solvents	Extraction characterization and gummy evaluation	Anthocyanins, DPPH, FRAP, color and antioxidant properties	Natural eutectic solvents produced anthocyanin-rich extracts with antioxidant activity, enabling intensely colored functional gummies	[133]
*Vitis vinifera* cv. Băbească Neagră grape pomace	Jelly candies with different pomace particle sizes and gelatin concentrations	Physicochemical, phytochemical, textural and sensory evaluation	Phenolics, antioxidant activity, color, hardness, cohesiveness and sensory acceptance	Grape pomace improved polyphenol content and purple color; extract <125 μm with 7 g gelatin showed the highest phenolics (156 mg GAE/g) and antioxidant activity (65.8%).	[134]
Apple pomace aqueous extract	Healthy jelly candies with apple pomace extract	Phytochemical, antioxidant, microbiological, storage and sensory evaluation	Phenolics, antioxidant activity, color, microbiological stability and sensory acceptance	Gummies showed high polyphenol content (8.25 mg GAE/g) and antioxidant capacity (142.03 mmol TE/g), with sensory scores > 4.70/5	[135]
Citrus peel extracts	Gelatin gummies containing red orange, blonde orange or lemon peel extracts	In vitro antioxidant and rheological evaluation	Phenolic profile, antioxidant activity, rheology and stability	Citrus peel extracts provided antioxidant activity; grafting polyphenols onto gelatin produced gummies with sustained antioxidant and rheological properties during storage	[136]
Orange juice by-products	Pectin jelly candies with hydroalcoholic or enzymatic extracts	Physicochemical evaluation + in vitro digestion	Phenolics, flavonoids, texture, color and bioaccessibility	Both extracts maintained technological properties and achieved high phenolic bioaccessibility (>90%), supporting pectin as an effective delivery matrix	[137]
Orange peel extract	Gummies with 7.5 or 15% ultrasound-assisted extract	Physicochemical evaluation + in vitro gastrointestinal digestion	Polyphenol profile, antioxidant activity and bioaccessibility	Hesperidin and narirutin were predominant; total polyphenol bioaccessibility reached 97.02% (7.5%) and 80.30% (15%)	[138]
Coffee husk extract	Healthy gummy jelly with different gelatin and honey levels	Physicochemical and sensory evaluation	Color, water activity, texture and sensory acceptance	20 g gelatin + 90 g honey achieved the highest overall acceptance (7.43); higher gelatin reduced sensory acceptance	[139]
Coffee silverskin extract	Gummy candies with 1–4% coffee silverskin extract	Chemical, physical, microbiological, structural and sensory evaluation during 120-day storage	Phenolics, antioxidant activity, quality, texture, structure and sensory properties	Coffee silverskin improved bioactive content, antioxidant activity and physical/sensory quality; benefits were maintained during storage	[140]
Red onion extract	Antioxidant gummy jelly with increasing extract concentrations	Physicochemical, antioxidant and sensory evaluation	Antioxidant activity, color, texture and sensory acceptance	Onion extract increased antioxidant potential; the developed gummies showed good sensory acceptance and potential as a functional confectionery	[141]
Surinam cherry and spine gourd fruit extracts	Jelly candies containing fruit extracts	*C. elegans* anthelmintic assay + physicochemical, microbial and sensory evaluation	Anthelmintic activity, texture, color, pH, water activity, stability and sensory acceptance	Both gummies showed anthelmintic activity; Surinam cherry candy showed greater efficacy, with acceptable quality maintained during storage	[142]
Apple pomace + grape pomace	Jelly candies with agro-residues and sucrose alternatives	Physicochemical, antioxidant and glycemic-related evaluation	Phenolics, antioxidant activity, sugars and glycemic response	Agro-residues and sucrose substitutes improved nutritional functionality and reduced predicted glucose-spike potential	[143]
Quince + sea buckthorn	Jelly candies enriched with fruit ingredients	Physicochemical, phytochemical and sensory evaluation	Phenolics, antioxidant activity, color, texture and sensory properties	Fruit incorporation enhanced bioactive content and antioxidant capacity while maintaining acceptable technological and sensory properties	[144]
Apple and beetroot pomace flour	Jelly candies with pomace flour	Physicochemical, nutritional and antioxidant evaluation	Phenolics, antioxidant activity, color, texture and nutritional composition	Pomace flour increased phenolic content, antioxidant activity and nutritional value, demonstrating potential for confectionery upcycling	[145]
Rosemary extract	Jelly candies with fructan fibers and stevia	Physicochemical, antioxidant and sensory evaluation	Antioxidant activity, phenolics, color, texture and sensory properties	Rosemary extract provided antioxidant protection; fructan fibers and stevia enabled a reduced-sugar functional gummy formulation	[146]
Purple basil anthocyanins	Gelatin gummies containing alginate–carrageenan emulgel beads	Physicochemical, stability and antioxidant evaluation	Anthocyanin stability, color, texture and antioxidant activity	Encapsulation improved anthocyanin protection and stability, enabling incorporation of purple basil pigments into gelatin gummies	[147]
Leafy vegetables	Free-sugar jelly with leafy vegetable extracts	Physicochemical, nutritional and sensory evaluation	Nutritional composition, phenolics, antioxidant activity and sensory acceptance	Vegetable incorporation produced functional, free-sugar jellies with enhanced nutritional and antioxidant properties	[148]
Lotus (*Nelumbo nucifera*) petals	Gummy jelly enriched with lotus petal bioactive compounds	Physicochemical, antioxidant and sensory evaluation	Phenolics, antioxidant activity, color, texture and sensory acceptance	Lotus petal compounds enhanced antioxidant properties while maintaining acceptable gummy characteristics	[149]
Globe amaranth + betel leaf extract	Gummy candy containing *Gomphrena globosa* and *Piper betle* extracts	Physicochemical and sensory evaluation	Color, texture, moisture and sensory acceptance	Betel leaf extract altered physicochemical properties while providing a plant-based source of bioactive compounds; formulations remained sensorially acceptable	[150]
*Lysiphyllum strychnifolium* leaf extract	Gummy jelly enriched with leaf extract	Antioxidant and α-glucosidase inhibition assays	Phenolics, antioxidant activity and α-glucosidase inhibition	Extract incorporation increased antioxidant and α-glucosidase inhibitory activities, supporting potential antidiabetic functionality	[151]
Hibiscus extract	Jelly candy containing anthocyanin-loaded microparticles	In vitro release + gummy evaluation	Anthocyanin retention, release and stability	Ionic-gelation encapsulation improved anthocyanin protection and controlled release in the jelly matrix	[152]
*Teucrium montanum* extract	Spray-dried CMC delivery system for plant-based confectionery	Physicochemical and bioactive characterization	Phenolics, antioxidant activity, encapsulation efficiency and stability	Spray drying generated stable CMC-based systems suitable for incorporating plant extracts into confectionery products	[153]
Hydrolyzed hemp (*Cannabis sativa*)	Nutraceutical gummy candy	Physicochemical, antioxidant and antihypertensive evaluation	Phenolics, antioxidant activity and ACE-inhibitory potential	Hemp hydrolysate provided antioxidant and potential antihypertensive functionality in a nutraceutical gummy matrix	[154]
*Withania somnifera* + *Valeriana officinalis*	Nutraceutical gummies	Rat forced-swim test	Immobility time and antidepressant-related activity	Gummies containing the botanical combination showed antidepressant-like effects in the forced-swim model	[155]
*Lactobacillus reuteri* + lemongrass essential oil	Acacia-gum candy with probiotic and essential oil	Storage stability study	Probiotic survival, physicochemical properties and stability	*L. reuteri* survived storage in the acacia-gum matrix, although viability decreased over time; formulation provided a potential probiotic confectionery vehicle	[156]
Fruit/vegetable ingredients and dietary fiber	Nutritionally improved jelly candies	Nutritional, physicochemical and sensory evaluation	Nutritional composition, texture, color and sensory acceptance	Reformulation improved the nutritional profile while maintaining acceptable technological and sensory characteristics	[157]
Pomegranate + beetroot	Pomegranate–beetroot gummies	Physicochemical, nutritional and sensory evaluation	Phenolics, antioxidant activity, color, texture and sensory acceptance	Pomegranate and beetroot incorporation enhanced bioactive and antioxidant potential, producing a healthier confectionery product	[158]
Pomegranate juice (*Punica granatum* cv. Mollar de Elche)	Jelly candies based on pomegranate juice	Physicochemical and consumer acceptance evaluation	Phenolics, antioxidant activity, color, texture and consumer acceptance	Pomegranate juice produced gummies with high bioactive content and antioxidant capacity and good consumer acceptance	[159]
Resveratrol	Gummies with free or encapsulated resveratrol	Consumer acceptance + product evaluation	Sensory acceptance, liking and purchase-related responses	Encapsulation improved the feasibility of incorporating resveratrol into gummies; consumer acceptance was generally favorable, with formulation effects on sensory responses	[160]
BRS Violeta grape juice	Jelly candy with grape juice, gelatin, honey and agar	Anthocyanin through HPLC-MS with RATA + sensory	Anthocyanins, retention and sensory acceptance	41% of juice anthocyanins were retained; delphinidin derivatives predominated and the candy showed satisfactory acceptance	[161]
Wild blueberry powder	Gummy product prepared with freeze-dried blueberry powder	Storage at 4.4 and 21 °C for 8 weeks	Anthocyanins, polyphenolics and polymeric color	Anthocyanin retention decreased during storage, reaching 43% at 4.4 °C and 51% at 21 °C; chlorogenic acid and flavonols were more stable	[162]
Cryoconcentrated blueberry juice	Commercial gelatin, aerated gelatin, gummy and aerated gummy hydrogels	Physicochemical, textural and storage evaluation	Polyphenols, anthocyanins, flavonoids and antioxidant stability	Gummy and gelatin gels showed the lowest bioactive degradation; gummies remained stable for up to ~21 days, supporting cryoconcentrated juice as a functional ingredient	[163]
Cryoconcentrated blueberry juice	Gelatin gels enriched with different CBJ concentrations	Rheological, textural and bioactive evaluation	Gel strength, hardness, rheology, polyphenols, anthocyanins and antioxidant activity	20% CBJ reinforced the gel structure, increasing gel strength, hardness, gumminess and chewiness while providing bioactive compounds	[164]
Cryoconcen-trated blue-berry juice	Aerated gelatin gels	Structural, color, bioactive and storage evaluation	Microstructure, color, phenolics, anthocyanins and antioxidant capacity	Cryoconcentrated juice enriched the gels with bioactives and antioxidant capacity while maintaining structural properties during storage	[165]
Black carrot concentrate powder	Gelatin-based model gummies	Physicochemical, texture, color and stability evaluation	Color, texture, phenolics and antioxidant activity	Black carrot concentrate interacted with gelatin and sucrose, improving appearance and taste; the optimized formulation contained 32.03% sucrose, 21% gelatin and 0.27% concentrate	[166]
Red beet extract powder	Gelatin gummies with different sucrose and gelatin levels	Physicochemical, texture, color, bioactive and stability evaluation	Color, texture, phenolics and antioxidant activity	Interactions among beet extract, gelatin and sucrose strongly affected color and texture; the optimized formulation contained 0.44% beet extract	[167]
*Opuntia ficus-indica* betalain extract	Gelatin gummies containing calcium-alginate betalain capsules	Rheological, structural, color and storage evaluation	Betalain stability, color, viscoelasticity and gel strength	Encapsulation produced vivid red-purple gummies and maintained betalain color stability during 30 days at 4 °C	[168]
Black carrot pomace + virgin olive oil	Functional jelly candies with 6.7–10.7 g pomace	Physicochemical, nutritional, textural and sensory evaluation	Texture, moisture, sugars, fiber, protein, fat and sensory acceptance	Increasing pomace reduced hardness and chewiness; the intermediate-pomace formulation achieved the highest overall acceptance (7.40/10), supporting by-product valorization	[169]
Carob flour (*Ceratonia siliqua*)	Gelatin-based gummies with different flour levels, roasting conditions and particle sizes	Nutritional, physicochemical, antioxidant, antidiabetic and sensory evaluation	Fiber, phenolics, D-pinitol, antioxidant activity, α-glucosidase inhibition, texture and acceptance	Carob flour improved nutritional and functional properties; moderate roasting enhanced antioxidant and antidiabetic activity, while fine milling improved texture and acceptance	[170]
Peanut skin phenolic extract	Pectin- and gelatin-based candies with 0.1–0.2 g/kg extract	Physicochemical evaluation and INFOGEST in vitro digestion	Phenolics, antioxidant activity and bioaccessibility	Phenolic bioaccessibility reached 29.88–32.46% in pectin candies and 25.94–30.16% in gelatin candies; both matrices retained considerable antioxidant capacity after digestion	[171]
Ginger powder	Vegan gummy candies with different ginger concentrations	Physicochemical, microbiological, textural and sensory evaluation during storage	Moisture, aw, pH, color, texture, microbial stability and sensory acceptance	Ginger powder modified color and texture while providing a plant-based functional ingredient; suitable formulations maintained acceptable quality during storage	[172]
Blackberry and elderberry anthocyanin extracts	Functional gummies containing freeze-dried berry extracts	Chemical, stability, plasma and cellular antioxidant/anti-inflammatory assays	Phenolics, anthocyanins, antioxidant activity, ROS, IL-6 and sensory properties	Elderberry extract showed higher phenolic and anthocyanin contents; berry gummies increased antioxidant potential, while elderberry extract more effectively reduced IL-6	[173]
Functional gummy formulations	Commercial/experimental functional gummies analyzed by metabolomics	High-resolution accurate-mass metabolomics	Phenolic compounds, flavonoids, metabolites and chemical profiles	Metabolomic analysis enabled the identification and profiling of bioactive compounds in functional gummies, supporting their chemical characterization and quality assessment	[174]
Spirulina and açaí	Gummy candies enriched with spirulina and açaí	Physicochemical, nutritional, antioxidant and sensory evaluation	Proximate composition, phenolics, antioxidant activity, color, texture and acceptance	Spirulina and açaí increased the functional and nutritional value of gummies while maintaining acceptable technological and sensory properties	[175]
Carotenoid extract from yellow coffee pulp	Gummies enriched with microwave-assisted coffee-pulp extract	Extraction optimization and gummy evaluation	Carotenoids, color, antioxidant activity, texture and sensory properties	Microwave-assisted extraction enabled the recovery of carotenoids from coffee pulp and their incorporation into gummies as a natural functional colorant	[176]
Apple pomace	Jelly candies containing apple pomace	Physicochemical, nutritional, antioxidant and sensory evaluation	Phenolics, antioxidant activity, fiber, color, texture and acceptance	Apple pomace improved the nutritional and antioxidant profile of jelly candies, supporting the valorization of fruit-processing waste within a circular-economy approach	[177]
Rose tea	Rose tea gummy jelly with 0–100% sucrose replacement by sucralose	Physicochemical, antioxidant and sensory evaluation	Reducing sugars, phenolics, flavonoids, DPPH, texture and acceptance	Sucrose replacement reduced reducing sugars; complete replacement produced the highest redness, whereas 50% replacement yielded the greatest hardness, gumminess and chewiness	[178]
Betanin	Gummy candy containing free betanin or betanin-loaded nanoliposomes	Stability, antioxidant and sensory evaluation	Betanin retention, antioxidant activity and sensory acceptance	Liposomal encapsulation improved betanin stability; betanin content and antioxidant activity were at least twice those of gummies containing free betanin, without negatively affecting acceptance	[179]

## Data Availability

No new data were created or analyzed in this study. Data sharing is not applicable to this article.

## Abbreviations

The following abbreviations are used in this manuscript:

CBD	Cannabidiol
DE	Dextrose equivalent
TPA	Texture profile analysis
FTIR	Fourier-transform infrared spectroscopy
SEM	Scanning electron microscopy
AFM	Atomic force microscopy
DSC	Differential scanning calorimetry
ROS	Reactive oxygen species
DPPH	2,2-diphenyl-1-picrylhydrazyl
FRAP	Ferric reducing antioxidant power
GAE	Gallic acid equivalents
TE	Trolox equivalents
FOS	Fructooligosaccharides
XOS	Xylooligosaccharides
PhG	Phenylethanoid glycoside
CMC	Carboxymethyl cellulose
ACE	Angiotensin-converting enzyme
HPLC-MS	High-performance liquid chromatography-mass spectrometry
RATA	Rate-All-That-Apply
CBJ	Cryoconcentrated blueberry juice
HPP	High pressure processing
PGP	Peach gum polysaccharide
LAOS	Large amplitude oscillatory shear
